# Age, brain region, and gene dosage-differential transcriptomic changes in *Shank3*-mutant mice

**DOI:** 10.3389/fnmol.2022.1017512

**Published:** 2022-10-12

**Authors:** Taesun Yoo, Ye-Eun Yoo, Hyojin Kang, Eunjoon Kim

**Affiliations:** ^1^Center for Synaptic Brain Dysfunctions, Institute for Basic Science (IBS), Daejeon, South Korea; ^2^Division of National Supercomputing, Korea Institute of Science and Technology Information (KISTI), Daejeon, South Korea; ^3^Department of Biological Sciences, Korea Advanced Institute of Science and Technology (KAIST), Daejeon, South Korea

**Keywords:** autism spectrum disorder, *Shank3*, age, cortex, hippocampus, striatum, gene dosage, RNA-seq

## Abstract

Shank3 is an abundant excitatory postsynaptic scaffolding protein implicated in various neurodevelopmental disorders, including autism spectrum disorder (ASD), Phelan-McDermid syndrome, intellectual disability, and schizophrenia. *Shank3*-mutant mice show various molecular, synaptic, and behavioral deficits, but little is known about how transcriptomic phenotypes vary across different ages, brain regions, and gene dosages. Here, we report transcriptomic patterns in the forebrains of juvenile and adult homozygous *Shank3*-mutant mice that lack exons 14–16 and also the prefrontal, hippocampal, and striatal transcriptomes in adult heterozygous and homozygous *Shank3*-mutant mice. The juvenile and adult mutant transcriptomes show patterns opposite from and similar to those observed in ASD (termed reverse-ASD and ASD-like patterns), respectively. The juvenile transcriptomic changes accompany synaptic upregulations and ribosomal and mitochondrial downregulations, whereas the adult transcriptome show opposite changes. The prefrontal, hippocampal, and striatal transcriptomes show differential changes in ASD-related gene expressions and biological functions associated with synapse, ribosome, mitochondria, and spliceosome. These patterns also differ across heterozygous and homozygous *Shank3*-mutant mice. These results suggest age, brain region, and gene dosage-differential transcriptomic changes in *Shank3*-mutant mice.

## Introduction

The Shank family proteins are postsynaptic scaffolding proteins that regulate excitatory synaptic development and function (Boeckers et al., 1999; Naisbitt et al., 1999; Sheng and Sala, 2001; Kim and Sheng, 2004; Sheng and Kim, 2011). Among the three known Shank family proteins, Shank2 and Shank3 have been associated with various brain disorders, including autism spectrum disorder (ASD), Phelan-McDermid syndrome, intellectual disability, and schizophrenia (Durand et al., 2007; Moessner et al., 2007; Gauthier et al., 2009; Berkel et al., 2010; Leblond et al., 2014; Phelan et al., 2022). Numerous studies on the functions of Shank2 and Shank3, including those using mouse genetic approaches, have provided substantial insights into the mechanisms underlying Shank2- or Shank3-related brain disorders (Bozdagi et al., 2010; Peca et al., 2011; Schmeisser et al., 2012; Won et al., 2012; reviewed in Sheng and Kim, 2000, 2011; Boeckers et al., 2002; Bourgeron, 2009; Grabrucker et al., 2011; Sala et al., 2015; Schmeisser, 2015; Monteiro and Feng, 2017; Mossa et al., 2017, 2018; De Rubeis et al., 2018; Eltokhi et al., 2018; Ey et al., 2020; Jung and Park, 2022). However, it remains unclear how the mechanistic deviations observed in *Shank3*-mutant mice differ by age or brain region under different gene-dosage conditions. Investigating gene-dosage effects is important, considering that there have been debates regarding whether heterozygous or homozygous *Shank3*-mutant mice provide a better model for human ASD conditions.

Here we compared transcriptomes of the forebrain regions of juvenile (P25) and adult (P60) Shank3-homozygous (HM) mice. In addition, we analyzed transcriptomic patterns in the prefrontal cortex (termed cortex hereafter), hippocampus, and striatum regions of adult (∼postnatal day 90 or P90) *Shank3* heterozygous (HT)- and homozygous (HM)-mutant mice lacking exons 14–16 (Shank3-HT/HM mice). We also compared these results with those previously reported from *Shank2*-mutant mice (Lee et al., 2021; Yoo et al., 2022). Our findings collectively indicate that there are age, brain region, and gene dosage-differential transcriptomes within and between *Shank2*- and *Shank3*-mutant mice, which may provide insight into altered biological functions and ASD-related/risk gene expression patterns.

## Materials and methods

### Animals

*Shank3*-mutant mice lacking exons 14–16 have been reported previously (Yoo et al., 2018, 2019) and were generated by Biocytogen. Mice were maintained at the mouse facility of the Korea Advanced Institute of Science and Technology (KAIST); they were fed *ad libitum* and maintained according to the Animal Research Requirements of KAIST.

### RNA-seq analysis

The abundance of the transcripts was quantified using Salmon (v1.1.0) (Patro et al., 2017) via a quasi-mapping approach with GC bias correction (–gcBias). The results were imported to R (v.4.1.3) using Tximport (Soneson et al., 2015) package, which was followed by the analysis of differential gene expression using R/Bioconductor DEseq2 (v1.30.1) (Love et al., 2014). Raw read counts were normalized to gene size and fitted to a negative binomial distribution. The *p* values were adjusted through multiple comparisons using Benjamini–Hochberg correction to obtain adjusted *p* values. Genes with adjusted *p* values less than 0.05 were considered as differentially expressed genes (DEGs). Volcano plots were generated using R ggplot2 (v.3.3.3) package.

Gene Set Enrichment Analysis (GSEA)^1^ (Subramanian et al., 2005) was used to determine whether WT and *Shank3*-mutant transcripts show significant enrichments for priori-defined gene sets. GSEA was performed using GSEAPreranked (gsea-3.0.jar) module on gene set collections downloaded from Molecular Signature Database (MSigDB) v7.4.^2^ GSEAPreranked was performed using the list of all genes expressed, ranked by the fold changes multiplied by the inverse of the *p* values with recommended default settings (1,000 permutations and a classic scoring scheme). The False Discovery Rate (FDR) was calculated to control for false positive outcomes by comparing the tails of the observed and null distributions derived from the 1,000 gene set permutations for a given Normalized Enrichment Score (NES). The gene sets with an FDR of less than 0.05 were considered as significant enrichment. Integration and visualization of the GSEA results were performed using the EnrichmentMap Cytoscape App (version 3.9.0) (Merico et al., 2010; Isserlin et al., 2014).

## Results

### DEG analysis and GSEA of P25-Shank3 and P60-Shank3 transcripts

**FIGURE 1:**
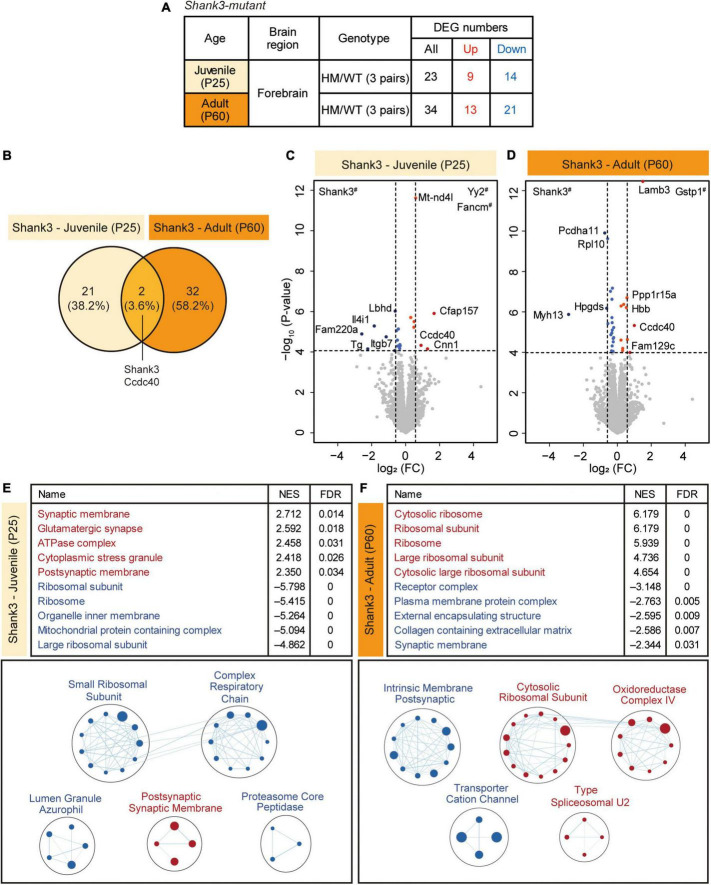
Differentially expressed gene (DEG) analysis and gene set enrichment analysis (GSEA) of P25-Shank3 and P60-Shank3 transcripts. **(A)** Summary of the DEGs from the forebrain of homozygous *Shank3*-mutant mice at P25 and P60 (P25-Shank3 and P60-Shank3 mice), compared with wild-type/WT mice at P25 and P60. DEGs were defined by transcript changes with adjusted *p* value < 0.05 (*n* = 3 mice for P25-WT, P25-Shank3, P60-WT, and P60-Shank3). **(B)** Venn diagrams showing DEGs that overlap between P25-Shank3 and P60-Shank3 mice. **(C,D)** Volcano plots showing DEGs from P25-Shank3 and P60-Shank3 mice. The DEGs (adjusted *p* value < 0.05) were further color-coded to indicate those with stronger fold changes (>1.5) (see Supplementary Table 2 for full results). Genes with # labels indicate those with *p* values beyond the indicated *p*-value ranges (*n* = 3 mice for P25-WT, P25-Shank3, P60-WT, and P60-Shank3). **(E,F)** GSEA of P25-Shank3 and P60-Shank3 transcripts were performed using the gene sets of the cellular component (CC) domain. The results are shown as lists of the top-five positively/negatively enriched gene sets (top; see Supplementary Table 3 for full results) and functional clustering of enriched gene sets, which was performed using the EnrichmentMap Cytoscape App (bottom). The sizes and colors of the circles in the EnrichmentMap results indicate the sizes of gene sets and the extents of positive/negative (red/blue) enrichments, respectively (*n* = 3 mice for P25-WT, P25-Shank3, P60-WT, and P60-Shank3).

To explore age-dependent transcriptomic changes in juvenile and adult *Shank3*-homozygous mutant mice lacking exons 14–16 (Yoo et al., 2018, 2019) at P25 and P60, respectively, we set out to perform RNA-Seq analysis of transcripts from mouse forebrain lacking the olfactory bulb (Figure 1A and Supplementary Table 1). The analysis of DEGs revealed relatively small sets of DEGs that were up- or downregulated in P25-Shank3 or P60-Shank3 mice (Figure 1A and Supplementary Table 2), and even smaller sets that overlapped between P25-Shank3 and P60-Shank3 mice (*Shank3* and *Ccdc40*) (Figure 1B). In the P25-Shank3 transcripts, the strongly upregulated DEGs included *Yy2* and *Fancm* and the strongly downregulated DEGs included *Shank3*, as shown by the volcano plot (Figure 1C). In the P60-Shank3 transcripts, the strongly upregulated DEGs included *Gstp1* and *Lamb3* and the strongly downregulated DEGs included *Shank3*, *Pcdha11*, and *Rpl10* (Figure 1D). CCDC40, a coiled-coil protein whose transcript levels are increased in both P25-Shank3 and P60-Shank3 transcripts, is known to regulate motile cilia function and left-right axis formation with implications in the primary ciliary dyskinesia (Becker-Heck et al., 2011). These results indicate that *Shank3* deletion in juvenile and adult mice is associated with relatively small sets of DEGs.

The scarcity of DEGs from P25-Shank3 and P60-Shank3 mice prompted us to apply GSEA. The results of GSEA performed using genes in the cellular component (CC) domain of the C5 gene sets indicated that P25-Shank3 transcripts were positively and moderately enriched for gene sets associated with synaptic functions (synaptic membrane, glutamatergic synapse, and postsynaptic membrane), as indicated by top-five most strongly enriched gene sets (Figure 1E, top and Supplementary Table 3). A similar conclusion was drawn from our functional clustering of positively enriched gene sets (postsynaptic membrane), which was performed using EnrichmentMap Cytoscape App (Figure 1E, bottom). P25-Shank3 transcripts were negatively and strongly enriched for gene sets associated with ribosomes (ribosomal subunit, ribosome, and large ribosomal subunit) and mitochondria (organelle inner membrane, mitochondrial protein-containing complex), as supported by the top-five gene sets and the EnrichmentMap results (Figure 1E). GSEA performed using the gene sets in the BP and MF domains of the C5 database yielded partly similar results; positive enrichments for synapse-related gene sets and negative enrichments for ribosome (translation)/mitochondria (electron transport, oxidative phosphorylation, and ATP synthesis)-related gene sets in the BP domain, and negative enrichments of P25-Shank3 transcripts for ribosome/mitochondria-related gene sets in the MF domain (Supplementary Figure 1 and Supplementary Table 3).

Gene set enrichment analysis of P60-Shank3 transcripts revealed strong positive enrichments for gene sets associated with ribosomes and mitochondria, as shown by the top-five gene sets and EnrichmentMap results (Figure 1F and Supplementary Table 3). In addition, P60-Shank3 transcripts were negatively but moderately enriched for synapse-related gene sets (receptor complex and synaptic membrane), as shown by the top-five gene sets and EnrichmentMap results (Figure 1F). GSEA using the gene sets in the BP and MF domains of the C5 database also yielded similar results; positive enrichments for ribosome/mitochondria-related gene sets in the BP domain, and positive enrichments for ribosome/mitochondria-related gene sets in the MF domain (Supplementary Figure 2 and Supplementary Table 3).

These findings indicate that Shank3 mice show age-dependent and nearly opposite transcriptomic patterns at juvenile and adult stages: Synaptic and ribosomal/mitochondrial genes are up- and downregulated, respectively, at P25, whereas opposite changes are observed at P60.

### ASD-related patterns in P25-Shank3 and P60-Shank3 transcripts

**FIGURE 2:**
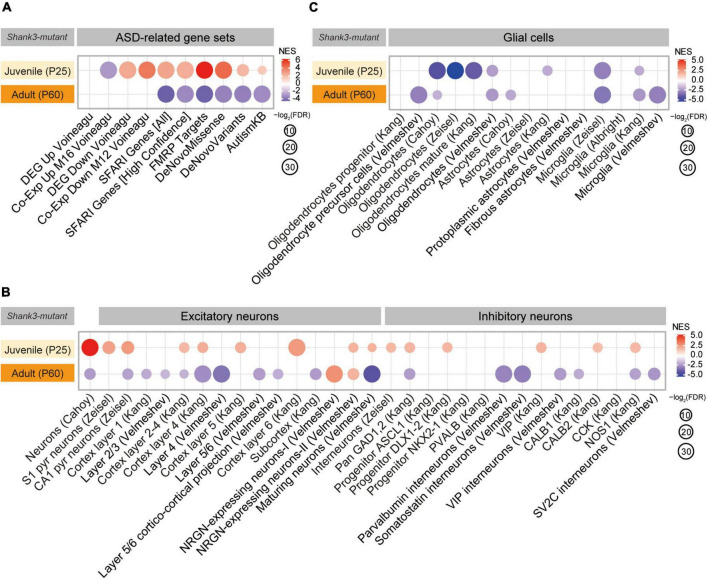
Autism spectrum disorder (ASD)-related patterns in P25-Shank3 and P60-Shank3 transcripts. **(A)** Gene set enrichment analysis (GSEA) results for P25- and P60-Shank3 transcripts relative to ASD-related gene sets that are upregulated (DEG Up Voineagu and Co-Exp Up M16 Voineagu) and downregulated (DEG Down Voineagu and Co-Exp Down M12 Voineagu) in ASD, as well as ASD-risk gene sets (SFARI Genes [All], SFARI Genes [High Confidence], FMRP Targets, DeNovoMissense, DeNovoVariants, and AutismKB) (*n* = 3 mice [P25-Shank3 and P60-Shank3]) (*n* = 3 mice [P25-Shank3 and P60-Shank3]). **(B)** GSEA results for P25- and P60-Shank3 transcripts for cell-type-specific gene sets (glutamate and GABA neurons) (*n* = 3 mice [P25-Shank3 and P60-Shank3]). **(C)** GSEA results for P25- and P60-Shank3 transcripts for cell-type-specific gene sets (glial cells) (*n* = 3 mice [P25-Shank3 and P60-Shank3]).

We next tested if P25-Shank3 and P60-Shank3 transcripts are enriched for ASD-related/risk gene sets. P25-Shank3 transcripts were negatively enriched for a gene set containing genes that are upregulated in ASD (Co-Exp Up M16 Voineagu) and positively enriched for gene sets containing genes that are downregulated in ASD (DEG Down Voineagu, and Co-Exp Down M12 Voineagu) (Voineagu et al., 2011; Werling et al., 2016; Figure 2A, Supplementary Figure 3, and Supplementary Table 4). In addition, P25-Shank3 transcripts were positively enriched for the SFARI gene set, which is usually downregulated in ASD, and other gene sets, such as FMRP Targets, DeNovoMissense, DeNovoVariants, and AutismKB (Figure 2A). These results suggest that P25-Shank3 transcripts display a transcriptomic pattern that is largely opposite to those observed in ASD. In contrast, P60-Shank3 transcripts were negatively enriched for ASD-risk gene sets (Figure 2A), and thus conformed to the pattern observed in ASD. The opposite enrichments of P25-Shank3 and P60-Shank3 transcripts for two gene sets (SFARI Genes [All] and FMRP Targets) involved ∼50% of the genes in each gene set and small correlative fold changes of co-up/down regulations (Supplementary Figure 4). These results suggest that P25- and P60-Shank3 transcripts show transcriptomic patterns that are largely opposite to each other, with a reverse-ASD pattern in juvenile stages converting to an ASD-like pattern in adult stages.

In GSEA performed using cell type-specific gene sets (Albright and Gonzalez-Scarano, 2004; Cahoy et al., 2008; Kang et al., 2011; Zeisel et al., 2015; Werling et al., 2016; Velmeshev et al., 2019, 2020; Supplementary Table 4), P25-Shank3 transcripts were positively enriched for neuron-related gene sets and negatively enriched for glia-related gene sets (Figures 2B,C). This pattern is largely opposite to that observed in ASD, which typically exhibits decreased neuronal/oligodendrocytic gene expression and increased astrocytic/microglial gene expression (Voineagu et al., 2011; Werling et al., 2016). The pattern in P60-Shank3 transcripts contrasted with that in P25-Shank3 transcripts by being negatively enriched for neuron-related gene sets, but resembled that in P25-Shank3 transcripts by being negatively enriched for oligodendrocyte/microglia-related gene sets (Figures 2B,C).

These GSEA results collectively suggest that P25- and P60-Shank3 transcripts display reverse-ASD and ASD-like transcriptomic patterns, respectively, based their enrichment patterns for gene sets that are up/downregulated in ASD, as well as those belonging to ASD-risk and cell type-specific gene sets.

### DEG analysis and GSEA of transcripts from the cortex, hippocampus, and striatum of Shank3-HT and Shank3-HM mice

**FIGURE 3:**
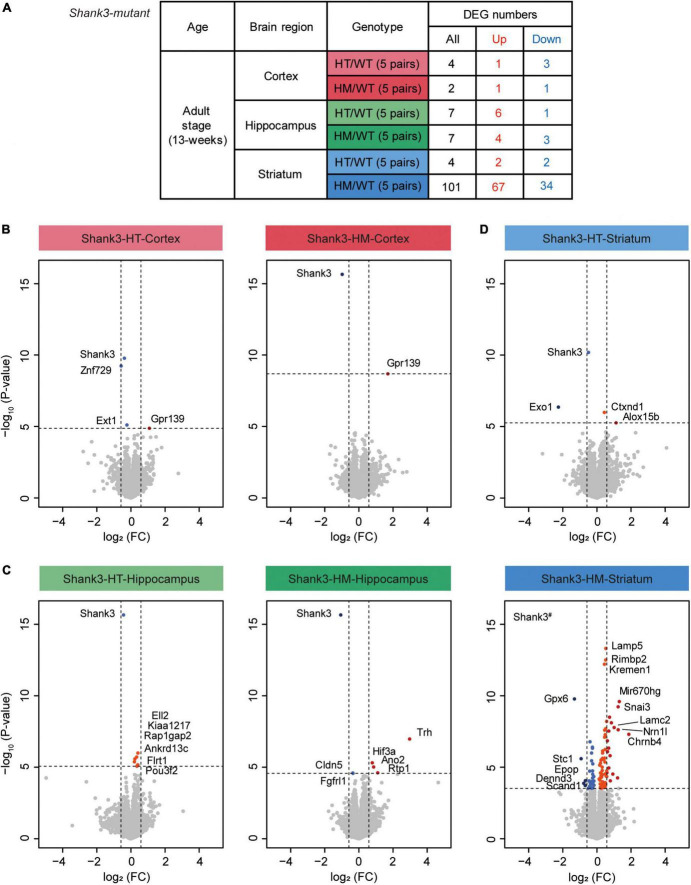
Differentially expressed gene (DEG) analysis of transcripts from the cortex, hippocampus, and striatum of Shank3-HT and Shank3-HM mice. **(A)** Outline of the DEGs from the cortex, hippocampus, and striatum of adult *Shank3*-heterozygous (HT) and *Shank3*-homozygous (HM) mutant mice, compared with WT mice. DEGs were defined by transcriptional changes with adjusted *p* value < 0.05 (*n* = 5 mice for cortex/hippocampus/striatum regions in WT, Shank3-HT, and Shank3-HM mice). **(B–D)** Volcano plots showing DEGs from the cortex, hippocampus, and striatum of adult *Shank3*-heterozygous (HT) and *Shank3*-homozygous (HM) mutant mice. The DEGs (adjusted *p* value < 0.05) were further color-coded to indicate those with stronger fold changes (>1.5). Shank3^#^ in the Shank3-HM volcano plot indicates a *p* value beyond the indicated *p*-value ranges (*n* = 5 mice for cortex/hippocampus/striatum regions in WT, Shank3-HT, and Shank3-HM mice).

We next tested if different brain regions and gene deletion dosages affect the transcriptomic patterns in adult (P90) heterozygous and homozygous *Shank3*-mutant mice (Shank3-HT and Shank3-HM mice, respectively; 5 mice per group) by performing RNA-Seq analyses of transcripts from the prefrontal cortex (termed cortex hereafter), hippocampus, and striatum (Figure 3A and Supplementary Table 5). These brain regional transcriptomes were well separated in a clustering analysis (Supplementary Figure 5).

All three brain regions of the Shank3-HT and Shank3-HM mice displayed small numbers of DEGs, except for the striatal region of Shank3-HM mice (Figure 3A). In volcano plots and the list of top DEGs, *Shank3* was identified in all six groups of downregulated DEGs (cortex/hippocampus/striatum of Shank3-HT/HM mice) (Figures 3B–D and Supplementary Table 6), indicating that the RNA-Seq results were generally reliable. The most prominent identified DEGs included *Znf729* (Shank3-HT cortical, downregulated), *Trh* (Shank3-HM hippocampal, upregulated), *Mir670hg* (Shank3-HM striatal, upregulated), and *Gpx6* (Shank3-HM striatal, downregulated). DAVID analysis of the striatal DEGs (*n* = 101) did not yield any significant biological GO term.

**FIGURE 4:**
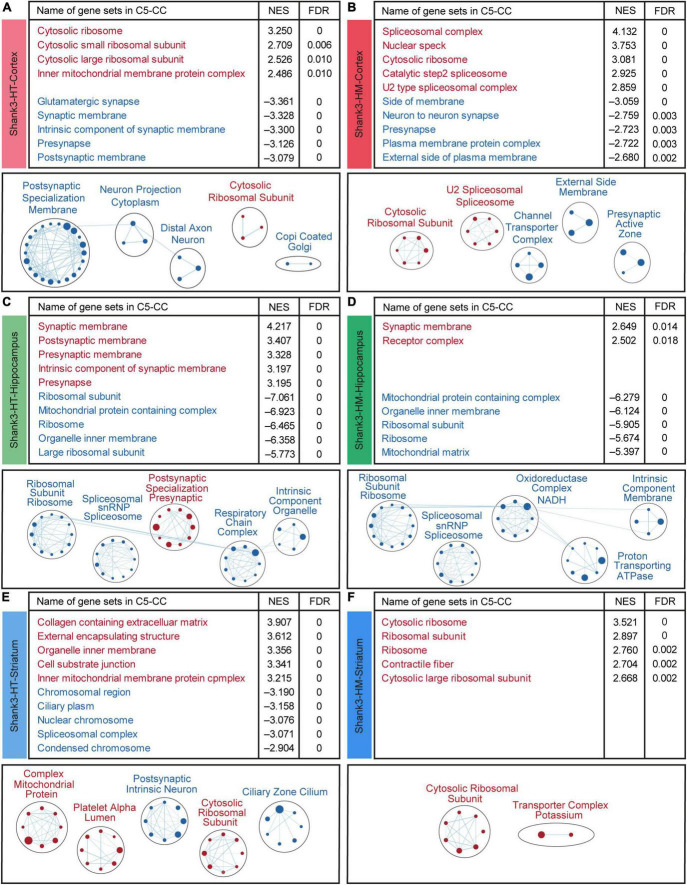
Gene set enrichment analysis (GSEA) of transcripts from the cortex, hippocampus, and striatum of Shank3-HT and Shank3-HM mice. **(A–F)** GSEA results obtained for cortical, hippocampal, and striatal Shank3-HT and Shank3-HM transcripts using the gene sets in the cellular component (CC) domain, as represented by the list of top-five positively/negatively enriched gene sets (top; see Supplementary Table 7 for full results) and functional clustering of enriched gene sets performed using the EnrichmentMap Cytoscape App (bottom). The sizes and colors of the circles in the EnrichmentMap results indicate the extents and directions (positive/negative; red/blue) of the enrichments, respectively (*n* = 5 mice for cortex/hippocampus/striatum regions in WT, Shank3-HT, and Shank3-HM mice).

We next performed GSEA to examine whether the cortical/hippocampal/striatal Shank3-HT and Shank3-HM transcripts were associated with specific biological functions. The cortical Shank3-HT transcripts were positively enriched for gene sets associated with ribosome/mitochondrial functions, as supported by the top-five gene sets and EnrichmentMap gene set clustering (Figure 4A and Supplementary Table 7). The cortical Shank3-HT transcripts were negatively enriched for gene sets associated with synaptic functions (Figure 4A). The cortical Shank3-HM transcripts were positively enriched for gene sets associated with spliceosomes and ribosomes, as supported by the top-five gene sets and EnrichmentMap gene set clustering, and negatively enriched for gene sets associated with synapses (neuronal synapse, presynapse, active zone) (Figure 4B). These results indicate that Shank3-HT and Shank3-HM cortical transcripts show similar upregulations of ribosome-related genes and downregulations of synapse-related genes.

The hippocampal Shank3-HT transcripts were positively enriched for synapse (pre/postsynaptic membrane)-related gene sets and negatively enriched for ribosome/mitochondria/spliceosome-related gene sets (Figure 4C). The hippocampal Shank3-HM transcripts were positively and weakly enriched for synapse-related gene sets and negatively enriched for ribosome/mitochondria, and spliceosome-related gene sets (Figure 4D). These results indicate that hippocampal Shank3-HT and Shank3-HM show similar patterns of synapse-related gene upregulation and ribosome/mitochondria- and spliceosome-related gene downregulation. Interestingly, these patterns are largely opposite those observed in the cortical Shank3-HT/HM transcripts (Figures 4A,B).

The striatal Shank3-HT transcripts were positively enriched for ribosome/mitochondria/ECM-related gene sets and negatively enriched for chromosome/spliceosome/cilia-related gene sets (Figure 4E). The striatal Shank3-HM transcripts were positively enriched for ribosome-related gene sets and did not exhibit any significant negative enrichment (Figure 4F). These results indicate that the striatal Shank3-HT and Shank3-HM transcript patterns are dissimilar to each other, except for the ribosome-related gene upregulation, and also dissimilar to the cortical and hippocampal patterns.

### ASD-related patterns in cortical, hippocampal, and striatal Shank3-HT and Shank3-HM transcripts

**FIGURE 5:**
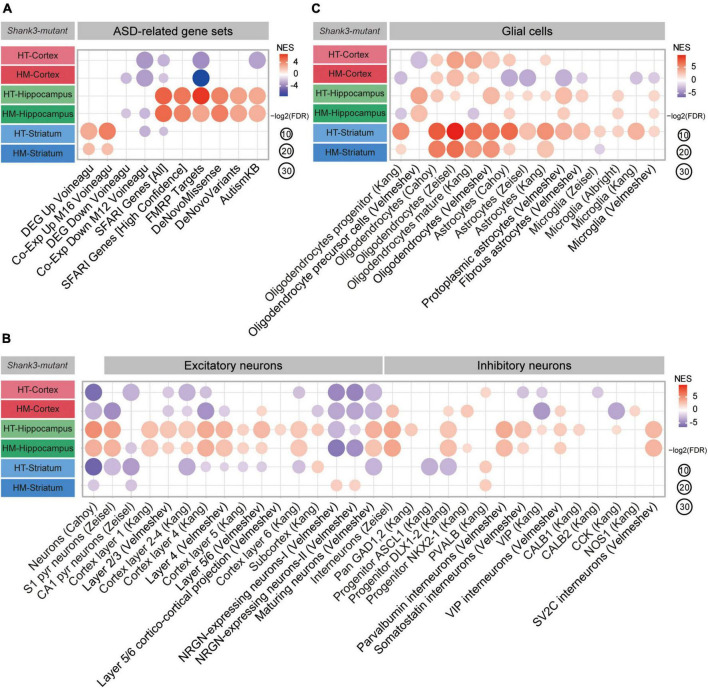
Autism spectrum disorder (ASD)-related patterns in the transcripts from the cortex, hippocampus, and striatum of Shank3-HT and Shank3-HM mice. **(A)** Gene set enrichment analysis (GSEA) results for the cortical, hippocampal, and striatal Shank3-HT and Shank3-HM transcripts: Enrichment patterns for ASD-related gene sets that are upregulated (DEG Up Voineagu and Co-Exp Up M16 Voineagu) or downregulated (DEG Down Voineagu and Co-Exp Down M12 Voineagu) in ASD, as well as for ASD-risk gene sets (SRARI Genes [All], SFARI Genes [High Confidence], FMRP Targets, DeNovoMissense, DeNovoVariants, and AutismKB) (*n* = 5 mice for cortex/hippocampus/striatum regions in WT, Shank3-HT, and Shank3-HM mice). **(B)** GSEA results for the cortical, hippocampal, and striatal Shank3-HT and Shank3-HM transcripts, indicating enrichment patterns for cell type-specific gene sets (glutamate and GABA neurons) (*n* = 5 mice for cortex/hippocampus/striatum regions in WT, Shank3-HT, and Shank3-HM mice). **(C)** GSEA results for the cortical, hippocampal, and striatal Shank3-HT and Shank3-HM transcripts, indicating enrichment patterns for cell type-specific gene sets (glial cells) (*n* = 5 mice for cortex/hippocampus/striatum regions in WT, Shank3-HT, and Shank3-HM mice).

We next tested whether the cortical, hippocampal, and striatal transcripts from Shank3-HT and Shank3-HM mice were enriched for ASD-related/risk gene sets. The cortical Shank3-HT transcripts were negatively enriched for the gene set downregulated in ASD (Co-Exp Down M12 Voineagu) (Voineagu et al., 2011; Werling et al., 2016) and negatively enriched for ASD-risk gene sets (SFARI Genes [All] and FMRP Targets) (Figure 5A, Supplementary Figure 6, and Supplementary Table 4). The cortical Shank3-HM transcripts showed similar ASD-like patterns.

In contrast, the hippocampal Shank3-HT and Shank3-HM transcripts displayed strong reverse-ASD patterns, as the transcripts were positively enriched for all six ASD-risk gene sets (SFARI Genes [All and High Confidence], FMRP Targets, DeNovoMissense, DeNovoVariants, and AutismKB) (Figure 5A). In contrast, the striatal Shank3-HT and Shank3-HM transcripts displayed ASD-like patterns: The transcripts were positively enriched for gene sets that are upregulated in ASD (DEG Up Voineagu, and Co-Exp Up M16 Voineagu), but no enrichment was observed for the ASD-risk gene sets (Figure 5A).

When GSEA was performed using cell type-specific gene sets, the results revealed that cortical Shank3-HT transcripts were negatively enriched for neuron-related gene sets, indicative of an ASD-like pattern. However, they were positively enriched for oligodendrocyte-related gene sets, which weakened the ASD-like pattern (Figures 5B,C). The cortical Shank3-HM transcripts were negatively enriched for neuron-related gene sets, which was similar to the ASD-like pattern seen in the cortical Shank3-HT transcripts. However, they were positively enriched for oligodendrocyte-related gene sets and negatively enriched for astrocyte/microglia-related gene sets, which weakened the ASD-like pattern.

The hippocampal Shank3-HT and Shank3-HM transcripts were positively enriched for neuron/oligodendrocyte-related gene sets, indicative of a reverse-ASD pattern. However, they were positively enriched for astrocyte/microglia-related gene sets, which weakened the reverse-ASD pattern (Figures 5B,C). The striatal Shank3-HT and Shank3-HM transcripts were negatively enriched for neuron-related gene sets and positively enriched for astrocyte/microglia-related gene sets, albeit to lesser extents in Shank3-HM transcripts, indicative of an ASD-like pattern. However, they were positively enriched for oligodendrocyte-related gene sets, which weakened the ASD-like pattern (Figures 5B,C).

These results from GSEA performed using ASD-related/risk and cell type-specific gene sets collectively suggest that cortical, hippocampal, and striatal Shank3-HT and Shank3-HM transcripts show both shared and distinct patterns wherein: (1) Shank3-HT and Shank3-HM show similar ASD-like patterns (limited gene dosage effects). (2) The cortex and striatum show ASD-like patterns, whereas the hippocampus shows a reverse-ASD pattern. (3) The ASD-like patterns in the cortex and striatum involve differential gene sets (the ASD-risk vs. ASD-related gene sets, respectively).

## Discussion

**FIGURE 6:**
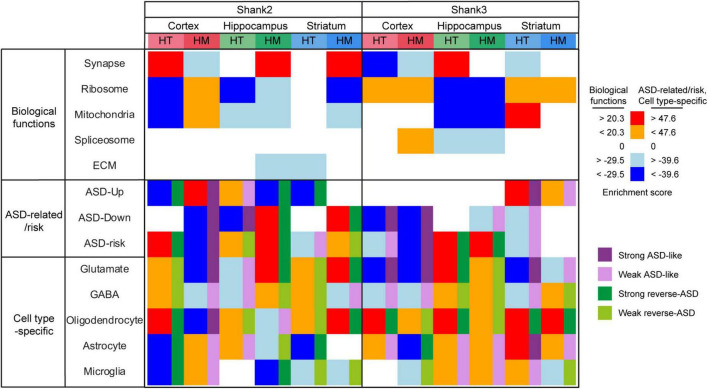
Summary of all gene set enrichment analysis (GSEA) results for Shank2-HT/HM and Shank3-HT/HM transcripts. To summarize the GSEA results for Shank2-HT/HM and Shank3-HT/HM transcripts in a single table, we selected the strongest gene-set cluster in the Cytoscape EnrichmentMap results (for gene sets associated with biological functions) or combined relevant gene sets [for autism spectrum disorder (ASD)-related/risk and single cell-type gene sets], and calculated comparative scores by summing the NES values of the gene sets in the indicated gene-set clusters (for biological functions) and by averaging the NES × *p* values of gene sets in the indicated gene-set groups (for ASD-related/risk and single-cell-specific gene sets) (see Supplementary Table 8 for the details).

**FIGURE 7:**
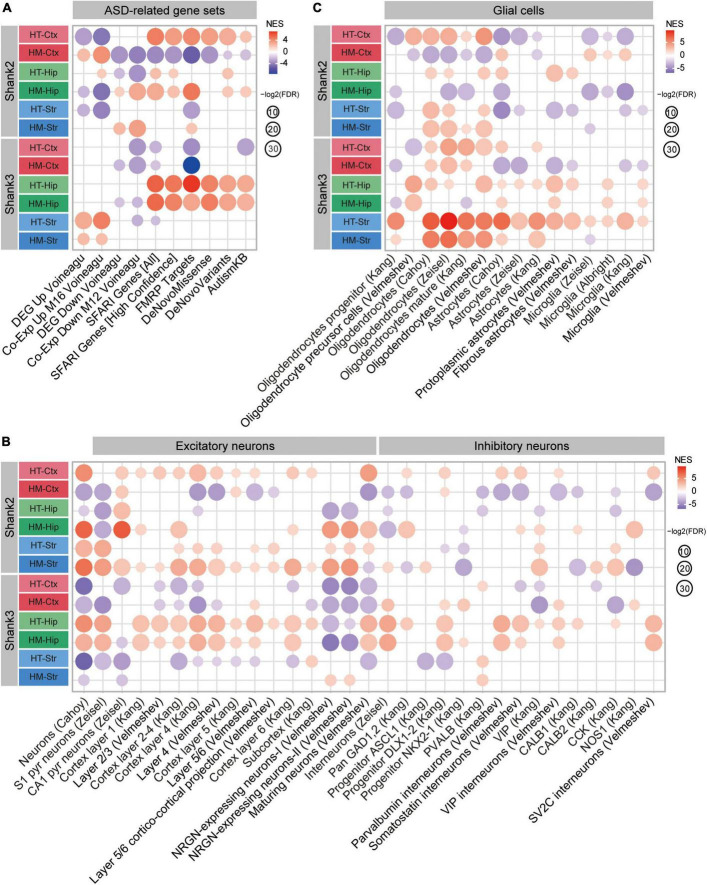
Summary of the gene set enrichment analysis (GSEA) results obtained using autism spectrum disorder (ASD)-related/risk and cell type-specific gene sets for Shank2-HT/HM and Shank3-HT/HM transcripts. **(A–C)** GSEA results for Shank2-HT/HM and Shank3-HT/HM transcripts obtained using ASD-related/risk and cell type-specific gene sets (Yoo et al., 2022; Figure 5) are combined here to enable better visualization of the similarities and differences.

Here we investigated transcriptomic changes in the prefrontal cortex, hippocampus, and striatum regions of adult Shank3-HT and Shank3-HM mice. In addition, we compared the transcriptomes from juvenile and adult Shank3-HM mice. The results point to age, brain region, and gene dosage-differential transcriptomic changes involving altered biological functions and expressions of ASD-related/risk genes in *Shank3*-mutant mice, which also differ from the overall patterns observed in *Shank2*-mutant mice (comparisons summarized in Figures 6, 7 and discussed below).

### Transcriptomic changes in *Shank3*-mutant mice

Juvenile (P25) and adult (P60) Shank3-HM mice showed largely contrasting transcriptomic changes in the forebrain. GSEA of P25 Shank3-HM transcripts revealed upregulation of synapse-related genes and downregulation of ribosome/mitochondria-related genes, corresponding to reverse ASD-like transcriptomic changes (Figures 1, 2). In contrast, P60 Shank3-HM transcripts showed downregulated synapse-related genes and upregulated ribosome/mitochondria-related genes, representing ASD-like transcriptomic patterns. These results suggest that these mice undergo a strong age-dependent transcriptomic change from a reverse-ASD pattern to an ASD-like pattern through the alteration of synaptic gene expression. A similar age-dependent change from a reverse-ASD to ASD-like pattern was previously reported in the mPFC region of Shank2-HM mice (exons 6–7), although the altered biological functions and ASD-related/risk genes were different (Lee et al., 2021). Despite this difference, the results from Shank2 and Shank3 mice collectively indicate at the minimum age-dependent transcriptomic inversion with respect to ASD-related/risk gene expressions.

Whether this age-dependent change in Shank3 forebrain transcriptomes correlates with altered neuronal and synaptic functions remains to be determined. Notably, however, the synaptic gene upregulations in the P25 *Shank3*-mutant forebrain correlate with the increased frequency of excitatory synaptic transmission in the juvenile *Shank3*-mutant mPFC (Yoo et al., 2019), although it remains unclear whether the adult *Shank3*-mutant mPFC would display suppressed excitatory transmission at P60 in line with the transcriptomic changes. It is possible that the early postnatal increase in excitatory synaptic function in *Shank3*-mutant mice may lead to an opposite change (excitatory synaptic depression) at adult stages. In support of this possibility, early and excessive excitatory synaptic functions have been shown to cause deleterious long-lasting effects in other mouse models of ASD, including Shank3B mice (exons 13–16 deletion) (Peixoto et al., 2016), Shank2 mice (Chung et al., 2019), and SynGAP1 mice (Clement et al., 2012, 2013; Aceti et al., 2015; reviewed in Chung et al., 2021).

Gene Set Enrichment Analysis of transcripts from the different brain regions of adult Shank3-HT and Shank3-HM mice revealed the following notable patterns (Figures 3–5): (1) The cortical, hippocampal, and striatal transcripts show distinctly altered biological functions and ASD-related/risk gene expression patterns. (2) Shank3-HT and Shank3-HM transcripts in a given brain region display largely similar patterns in biological functions and ASD-related/risk gene expression, suggesting that the gene dosage effect is small. (3) Synapse- and ribosome/mitochondria-related gene expression patterns frequently change in opposite directions consistently across all three brain regions. (4) Upregulated synapse-related genes and downregulated ribosome/mitochondria-related genes are frequently associated with reverse-ASD transcriptomic changes, and vice versa. (5) The hippocampal HT/HM transcripts display a reverse-ASD pattern, whereas the cortical and striatal HT/HM transcripts display ASD-like patterns. These patterns are partly similar to and distinct from those observed in *Shank2*-mutant mice (see below).

Whether these brain region-differential transcriptomic changes are associated with parallel changes in proteomes remains to be determined. However, a previous proteomic study on hippocampal and striatal postsynaptic density fractions from *Shank3*Δ*11^–/–^* mice reported enrichments of the differentially expressed proteins for actin- and synapse-related GO functions, respectively (Reim et al., 2017). This differs from our results in that hippocampal and striatal transcripts from Shank3-HM mice show enrichments for ribosome/mitochondria- and ribosome-related functions, respectively. The reason for the discrepancy could be that different *Shank3* exons were deleted in the two mouse lines.

### Comparison of *Shank2-* and *Shank3*-mutant transcriptomes

Differentially expressed gene analyses revealed an interesting difference between *Shank2*- and *Shank3*-mutant transcriptomes: More DEGs were identified in Shank2-HT/HM transcriptomes than in Shank3-HT/HM transcriptomes (Figure 3; Yoo et al., 2022). However, GSEA revealed strong enrichments of both *Shank2*- and *Shank3*-mutant transcriptomes for biological functions and ASD-related/risk gene sets (Figures 4, 5; Yoo et al., 2022). Therefore, *Shank2* deletion seems to induce two distinct types of transcriptomic changes: large changes in a small number of top genes that are readily detectable by DEG analyses, and small changes in a large number of genes that are readily detectable by GSEA. Meanwhile, *Shank3* deletion appears to induce mainly small changes in a large number of genes.

For *Shank2*- and *Shank3*-mutant transcriptomes, the GSEA results for biological functions and ASD-related/risk gene expressions reveal notable similarities and differences (see Figure 6 and Supplementary Table 8). Regarding similarities, we note that: (1) The three brain regions show distinct transcriptomic changes in both *Shank2*- and *Shank3*-mutant mice. (2) Synapse- and ribosome/mitochondria-related genes are frequently changed toward opposite directions in all three brain regions. (3) Upregulated synapse-related genes and downregulated ribosome/mitochondria-related genes are frequently associated with reverse ASD-like transcriptomic changes, and vice versa.

Regarding dissimilarities, we find that: (1) Shank2-HT/HM and Shank3-HT/HM transcripts show different gene dosage effects, in that Shank2-HT and Shank2-HM transcripts show largely opposite patterns with regard to altered biological functions and ASD-related/risk gene expressions, whereas Shank3-HT and Shank3-HM transcripts are largely similar in these aspects. (2) Stronger similarities across Shank2 and Shank3 mice are observed in HM conditions mice, whereas stronger dissimilarities are observed in HT conditions; i.e., Shank2-HM and Shank3-HM cortical transcripts show similar ASD-like patterns, and Shank2-HM and Shank3-HM hippocampal transcripts show similar reverse-ASD patterns, whereas Shank2-HT and Shank3-HT cortical transcripts, or Shank2-HT and Shank3-HT hippocampal transcripts, show opposite ASD-like patterns. (3) Opposite ASD-like patterns are observed in the striatal regions of Shank2-HT and Shank3-HT mice (reverse-ASD and ASD-like, respectively), similar to cortical and hippocampal regions, although the difference becomes less clear in Shank2/3-HM striatal transcripts, unlike cortical and hippocampal regions in which Shank2/3-HM transcripts become similar.

Additional similarities and differences between *Shank2*- and *Shank3*-mutant mice were also evident in detailed comparisons of ASD-related/risk GSEA patterns (Figure 7), as follows: (1) The changes observed in the ASD-related/risk gene expression patterns of Shank2-HT/HM transcripts involved both ASD-related and ASD-risk changes, whereas those in Shank3-HT/HM transcriptomes involved largely either ASD-related or ASD-risk changes. (2) The changes observed in neuronal gene expression patterns involved both excitatory and inhibitory neurons in both Shank2-HT/HM and Shank3-HT/HM transcripts. (3) Although ASD usually induces opposite changes in two groups of glial cell transcripts that are frequently observed in ASD (oligodendrocytic downregulations and astrocytic/microglial upregulations), we frequently observed exceptions to this (i.e., similar oligodendrocytic/astrocytic/microglial upregulations or downregulations) in both Shank2-HT/HM and Shank3-HT/HM transcriptomes (i.e., in that of Shank3-HT striatum).

Interpretations of the largely opposite changes in the expression patterns of synaptic genes and ribosomal/mitochondrial genes could differ in different brain regions and gene dosage conditions. For instance, the upregulation of synaptic genes in the Shank2-HM hippocampal transcriptome (Yoo et al., 2022), which coincides with the decreased synaptic transmission in the mutant hippocampus (Won et al., 2012), might reflect compensatory increases in synaptic gene expression. Meanwhile, the decreased synaptic transmission in *Shank2*-mutant mice might have suppressed ribosomal/mitochondrial gene expression to minimize energy production and expenditure (for protein synthesis) (Morita et al., 2015) in the absence of synaptic activity. In support of this possibility, synaptic activity has been functionally coupled with mitochondrial activity (Li et al., 2004; Vos et al., 2010; Sheng and Cai, 2012; Santini and Klann, 2014; Lee et al., 2018). In addition, ASD has been associated with mitochondrial deficits (Hollis et al., 2017; Frye, 2020; Rojas-Charry et al., 2021) and altered levels of ribosomal proteins in post-mortem brains and human neural progenitor cells (Lombardo, 2021).

Our observation that strong gene dosage differences are seen in Shank2 transcriptomes but not in Shank3 transcriptomes could reflect that *Shank2* heterozygous and homozygous deletions fall in a range where they could induce quantitively different disruptions of biological functions. The lack of dosage responses in the Shank3 transcriptomes could indicate that *Shank3* heterozygous deletion is sufficient to yield the full spectrum of phenotypic deficits. This might suggest the stronger impacts of *Shank3* mutations relative to *Shank2* mutations in animal models of ASD, and might be in line with the greater prevalence of *Shank3* mutations in ASD, relative to *Shank2* mutations (Leblond et al., 2014).

The current RNA-Seq results do not give clear answers on whether the observed transcriptomic changes represent molecular pathophysiology or responses that arise to compensate for the gene deletion. In addition, it would be difficult to functionally validate the identified biological functions and pathways, given the known and expected complexity of synaptic, ribosomal, and mitochondrial systems in different brain cell types. However, RNA-Seq analyses can provide unbiased clues on altered biological functions and hints on whether the overall transcriptomic changes in our systems mimic those observed in ASD (ASD-like vs. reverse-ASD). For instance, regardless of whether certain transcriptomic changes represent pathophysiology or responses, the absence of ASD-like transcriptomic changes indicate the absence or normalization of ASD-related phenotypes, i.e., at neuronal, synaptic, circuit, or behavioral levels. In addition, RNA-Seq analyses are useful in that they can be attempted in various *in vivo* contexts, including in different mouse ages, brain regions, gene dosages (heterozygous and homozygous), and pathological/rescue environments. The results may facilitate the design and data interpretation of future experiments. For instance, the results obtained in our setting suggest that *Shank3*- and *Shank2*-mutant mice may exhibit distinct gene dosage-related shifts in phenotypes (heterozygous vs. homozygous). In addition, other studies showed that RNA-Seq can be used to monitor ASD-related transcriptomic phenotypes when *Shank2*-mutant mice were treated with memantine at early postnatal stages, which corrected the synaptic and behavioral phenotypes at adult stages (Chung et al., 2019, 2021; Yoo et al., 2021).

The RNA-Seq results in the present study were obtained specifically from *Shank2*- and *Shank3*-mutant mouse lines lacking exons 6–7 and 14–16, respectively (Won et al., 2012; Yoo et al., 2018, 2019, 2021; Chung et al., 2019). Given that different mutations in the same Shank genes can lead to different mouse phenotypes (Sheng and Kim, 2000, 2011; Boeckers et al., 2002; Bourgeron, 2009; Grabrucker et al., 2011; Jiang and Ehlers, 2013; Sala et al., 2015; Schmeisser, 2015; Monteiro and Feng, 2017; Mossa et al., 2017, 2018; De Rubeis et al., 2018; Eltokhi et al., 2018; Ey et al., 2020), further work is warranted to examine whether convergent transcriptomic changes are be observed in additional *Shank2*- and *Shank3*-mutant mouse lines.

In summary, our results, together with the previous transcriptomic results from Shank2 mice, indicate that *Shank2* and *Shank3* deletions lead to age-, brain region-, and gene dosage-differential transcriptomic changes associated with altered biological functions and ASD-related/risk gene expression patterns. These results provide unbiased clues on the mechanisms underlying the ASD-related phenotypes in *Shank2/3*-mutant mice and will be useful in designing future experiments using these mice and interpreting the results.

## Data availability statement

The datasets presented in this study can be found in online repositories. The raw RNA-Seq results are available as GSE201621 (Shank3 brain regions), GSE201853 (Shank3 juvenile and adult forebrains), and GSE201854 (GSE201621 + GSE201853) at GEO (Gene Expression Omnibus), NCBI (National Center for Biotechnology Information).

## Ethics statement

The animal study was reviewed and approved by the Committee of Animal Research at KAIST (KA2020-93).

## Author contributions

TY, Y-EY, and EK designed the experiments. TY, Y-EY, and HK performed RNA-Seq analyses. HK and EK wrote the manuscript. All authors contributed to the article and approved the submitted version.

## Footnotes

1
http://software.broadinstitute.org/gsea

2
http://software.broadinstitute.org/gsea/msigdb

## Supplementary material

Supplementary Figure 1 Gene set enrichment analysis (GSEA) of P25-Shank3 transcripts using the gene sets in the biological process (BP) and molecular function (MF) domains. **(A,B)** Results of GSEA performed for P25-Shank3 forebrain transcripts using gene sets in the BP and MF domains, represented by the list of top-five positively/negatively enriched gene sets (top; see Supplementary Table 3 for full results) and functional clustering of enriched gene sets performed using the EnrichmentMap Cytoscape App (bottom) (*n* = 3 mice [P25-Shank3]).

Supplementary Figure 2 Gene set enrichment analysis (GSEA) for P60-Shank3 transcripts using the gene sets in the biological process (BP) and molecular function (MF) domains. **(A,B)** Results of GSEA for P60-Shank3 forebrain transcripts performed using gene sets in the BP and MF domains, represented by the list of top-five positively/negatively enriched gene sets (top; see Supplementary Table 3 for full results) and functional clustering of enriched gene sets performed using the EnrichmentMap Cytoscape App (bottom) (*n* = 3 mice [P60-Shank3]).

Supplementary Figure 3 Autism spectrum disorder (ASD)-related patterns in P25-Shank3 and P60-Shank3 transcripts. **(A–C)** The GSEA results in Figure 2 are shown here again with the insignificantly enriched gene sets indicated by square dots, together with the significant enrichments indicated by circular dots, to show that the insignificant enrichments have generally smaller NES scores compared with those of significant enrichments.

Supplementary Figure 4 Individual gene expression patterns for the opposite enrichments of P25-Shank3 and P60-Shank3 forebrain transcripts for two select autism spectrum disorder (ASD)-risk gene sets. **(A,B)** The opposite enrichments of P25-Shank3 and P60-Shank3 forebrain transcripts for two select ASD-risk gene sets (SFARI Genes [All] and FMRP Targets) were mediated by ∼50% of the genes in the gene sets **(A)** and are further supported by correlative fold changes for co-up/down regulations **(B)** [*n* = 3 mice (P25-Shank3 and P60-Shank3), Pearson test].

Supplementary Figure 5 Distinct clustering of three brain regional transcriptomes from Shank3-HT/HM mice. Clustering of cortical, hippocampal, and striatal transcriptomes from Shank3-WT, Shank3-HT, and Shank3-HM mice shown by heatmaps (13 weeks; male; *n* = 5 mice [WT], 5 [HT], and 5 [HM]).

Supplementary Figure 6 ASD-related patterns in the transcripts from the cortex, hippocampus, and striatum of Shank3-HT and Shank3-HM mice. **(A–C)** The GSEA results in Figure 5 are shown here again with the insignificantly enriched gene sets indicated by square dots, together with the significant enrichments indicated by circular dots, to show that the insignificant enrichments have generally smaller NES scores compared with those of significant enrichments.
