# Supplementary figures and images for: Age, brain region, and gene dosage-differential transcriptomic changes in Shank3-mutant mice
Source: Front Mol Neurosci. 2022 Oct 12;15:1017512. doi: 10.3389/fnmol.2022.1017512 (PMC9597470; doi:10.3389/fnmol.2022.1017512)

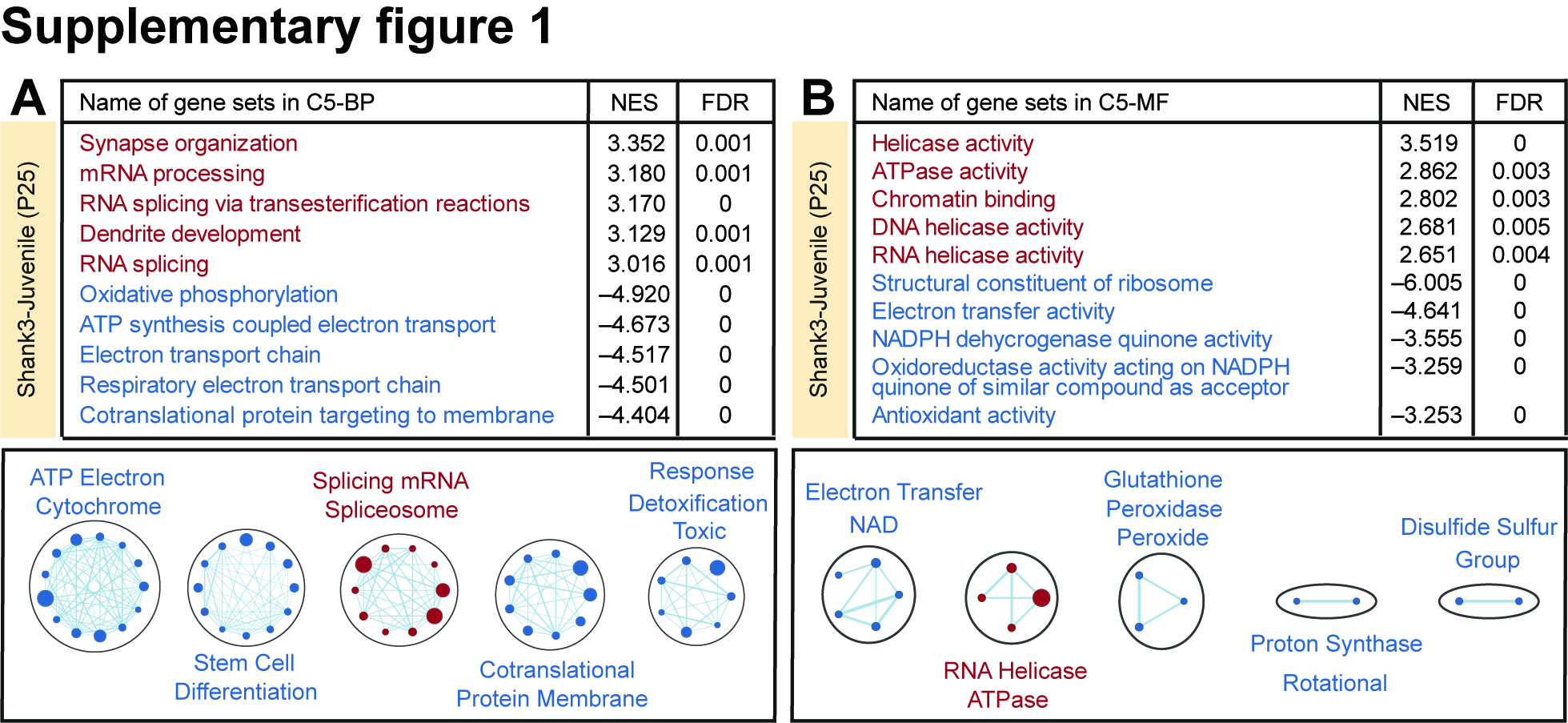

Supplement: Supplementary Figure 1 — Gene set enrichment analysis (GSEA) of P25-Shank3 transcripts using the gene sets in the biological process (BP) and molecular function (MF) domains. (A,B) Results of GSEA performed for P25-Shank3 forebrain transcripts using gene sets in the BP and MF domains, represented by the list of top-five positively/negatively enriched gene sets (top; see Supplementary Table 3 for full results) and functional clustering of enriched gene sets performed using the EnrichmentMap Cytoscape App (bottom) (n = 3 mice [P25-Shank3]). [file Image_1.TIF]

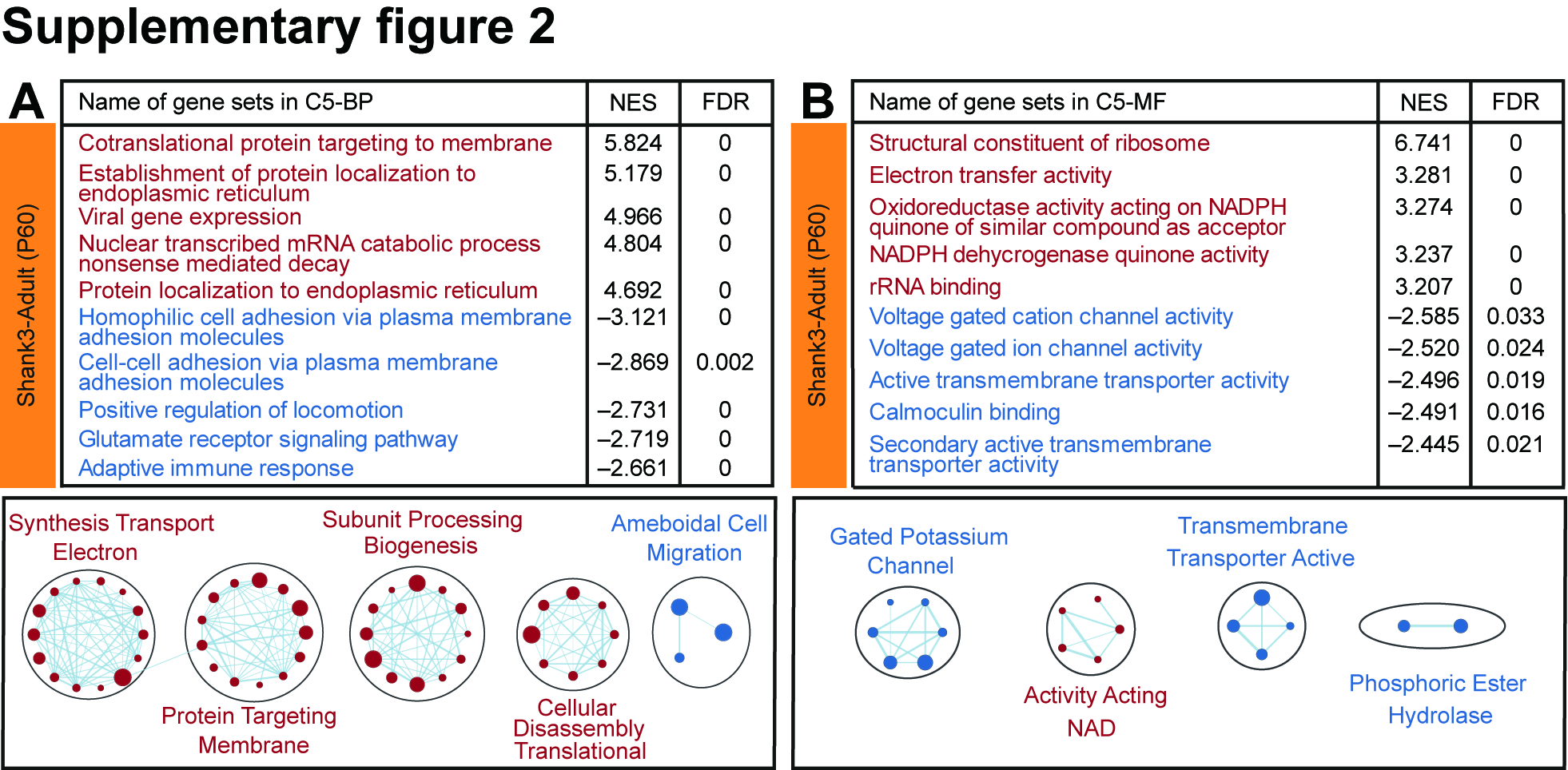

Supplement: Supplementary Figure 2 — Gene set enrichment analysis (GSEA) for P60-Shank3 transcripts using the gene sets in the biological process (BP) and molecular function (MF) domains. (A,B) Results of GSEA for P60-Shank3 forebrain transcripts performed using gene sets in the BP and MF domains, represented by the list of top-five positively/negatively enriched gene sets (top; see Supplementary Table 3 for full results) and functional clustering of enriched gene sets performed using the EnrichmentMap Cytoscape App (bottom) (n = 3 mice [P60-Shank3]). [file Image_2.TIF]

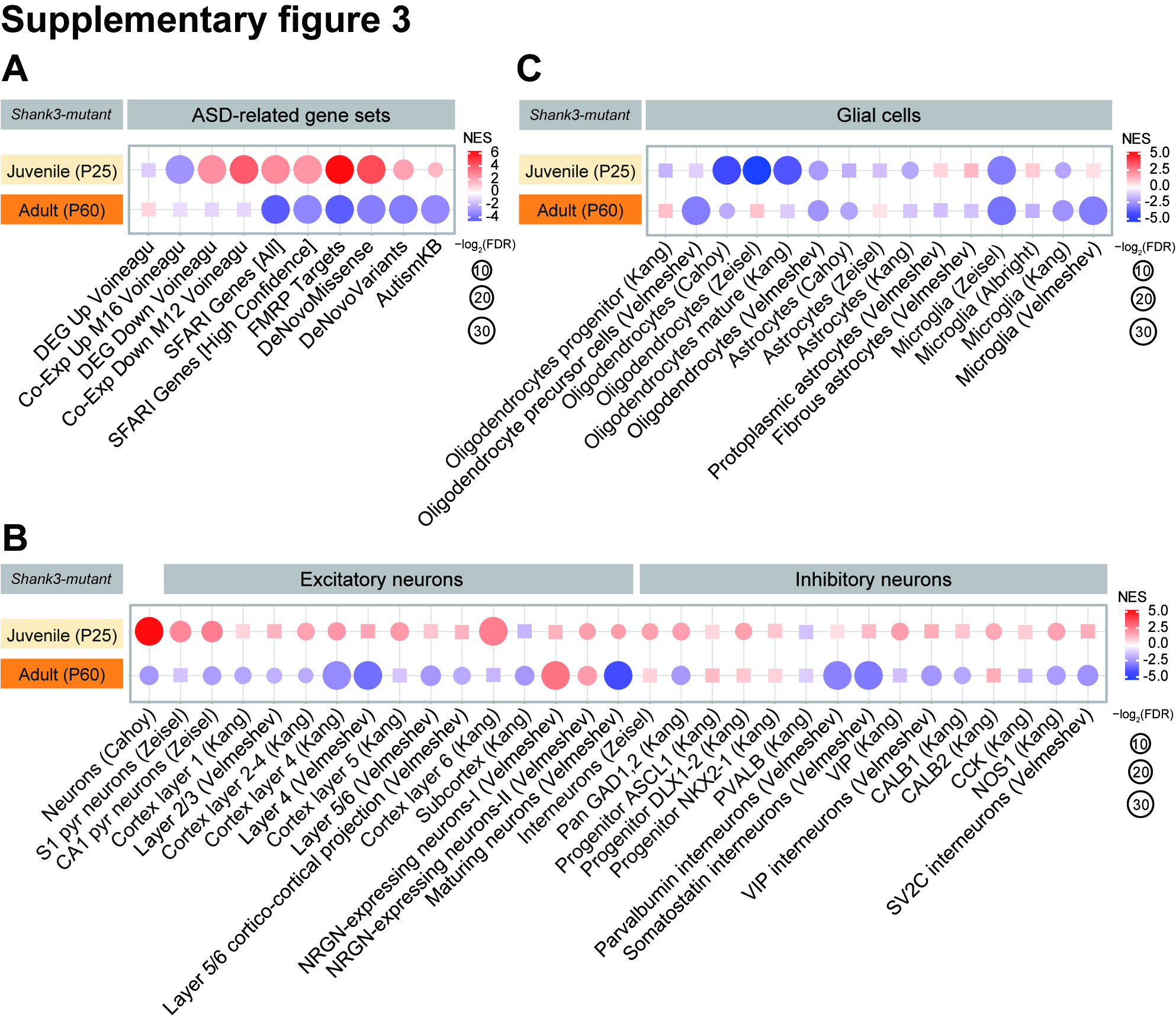

Supplement: Supplementary Figure 3 — Autism spectrum disorder (ASD)-related patterns in P25-Shank3 and P60-Shank3 transcripts. (A–C) The GSEA results in Figure 2 are shown here again with the insignificantly enriched gene sets indicated by square dots, together with the significant enrichments indicated by circular dots, to show that the insignificant enrichments have generally smaller NES scores compared with those of significant enrichments. [file Image_3.TIF]

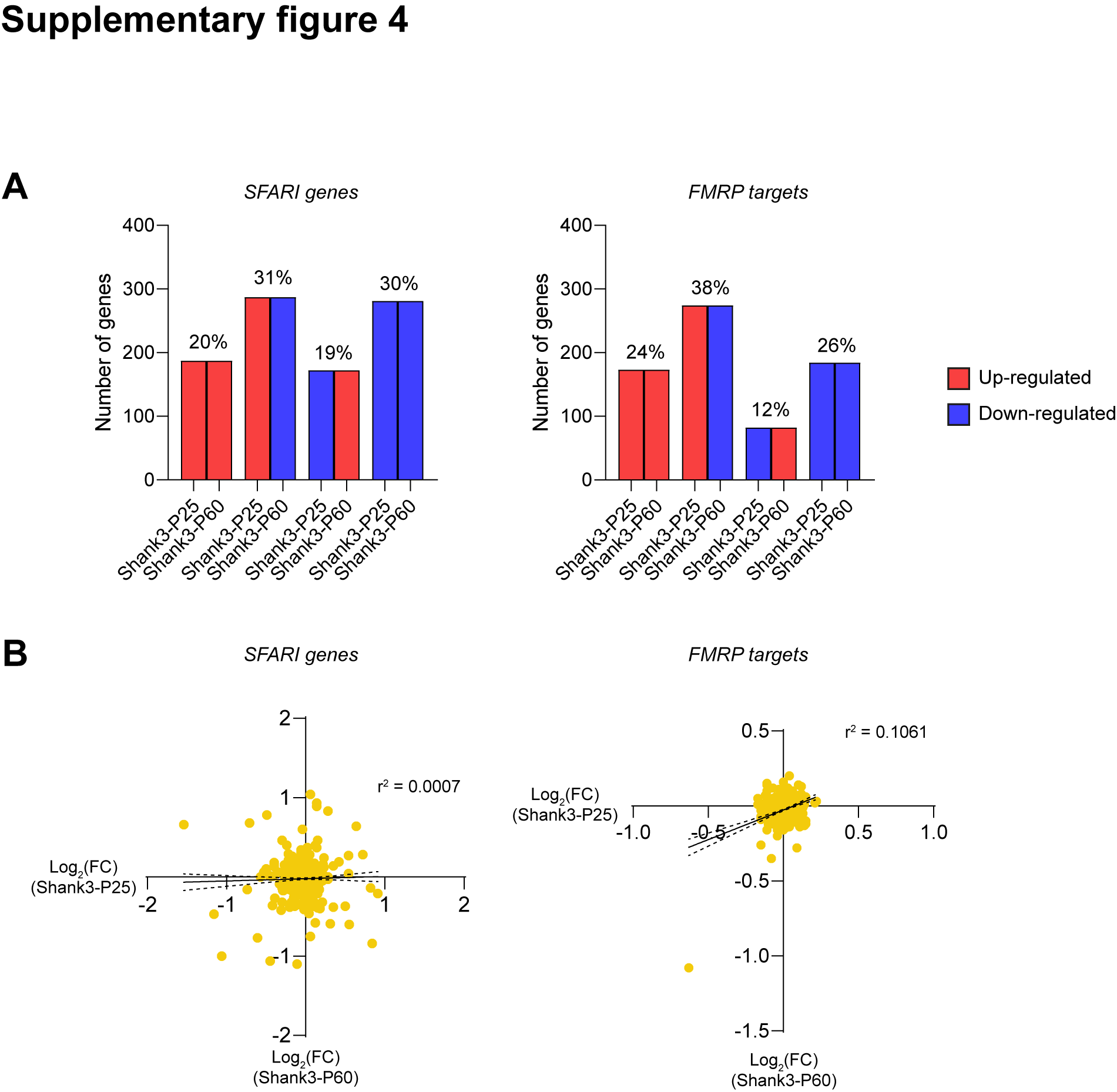

Supplement: Supplementary Figure 4 — Individual gene expression patterns for the opposite enrichments of P25-Shank3 and P60-Shank3 forebrain transcripts for two select autism spectrum disorder (ASD)-risk gene sets. (A,B) The opposite enrichments of P25-Shank3 and P60-Shank3 forebrain transcripts for two select ASD-risk gene sets (SFARI Genes [All] and FMRP Targets) were mediated by ∼50% of the genes in the gene sets (A) and are further supported by correlative fold changes for co-up/down regulations (B) [n = 3 mice (P25-Shank3 and P60-Shank3), Pearson test]. [file Image_4.TIF]

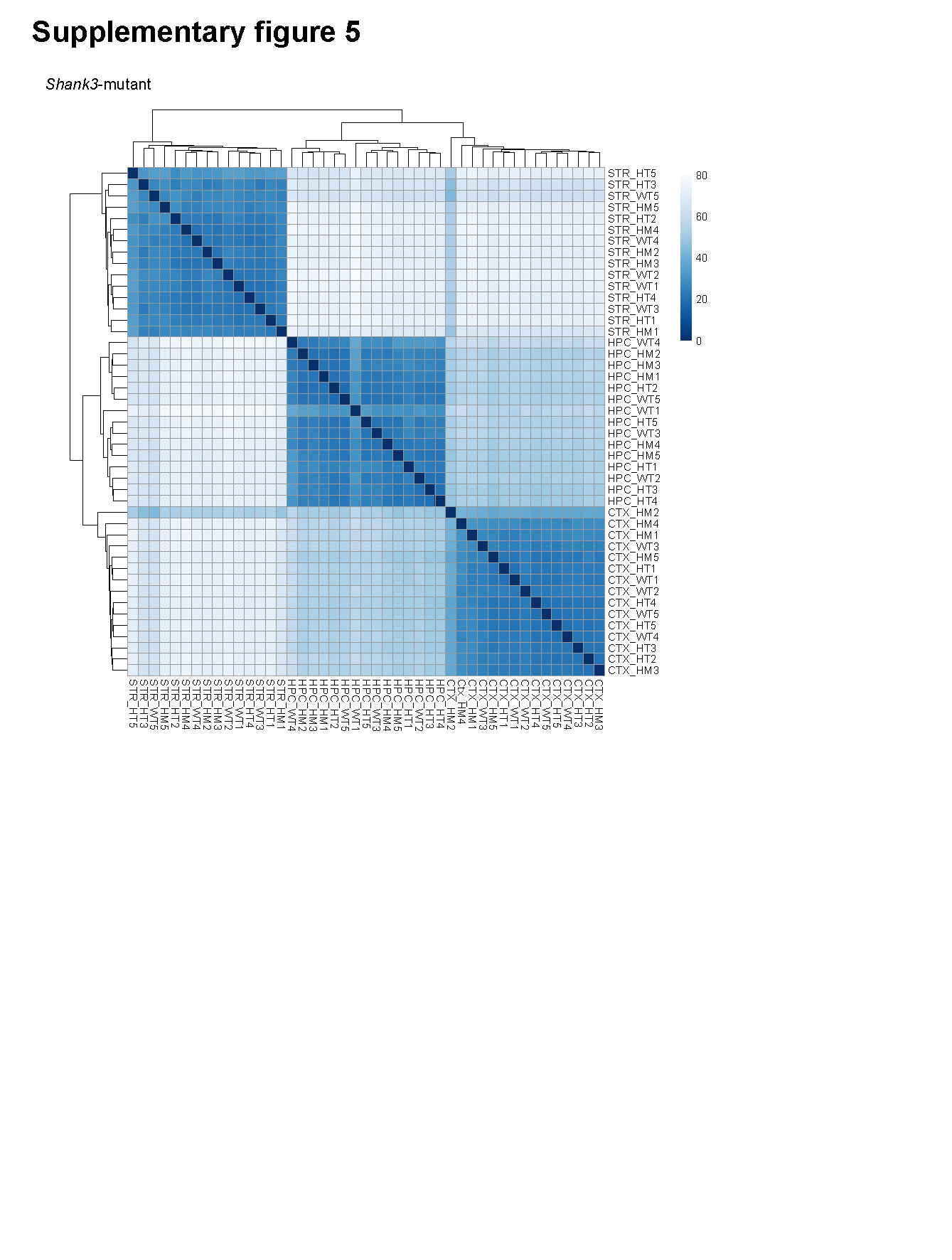

Supplement: Supplementary Figure 5 — Distinct clustering of three brain regional transcriptomes from Shank3-HT/HM mice. Clustering of cortical, hippocampal, and striatal transcriptomes from Shank3-WT, Shank3-HT, and Shank3-HM mice shown by heatmaps (13 weeks; male; n = 5 mice [WT], 5 [HT], and 5 [HM]). [file Image_5.tiff]

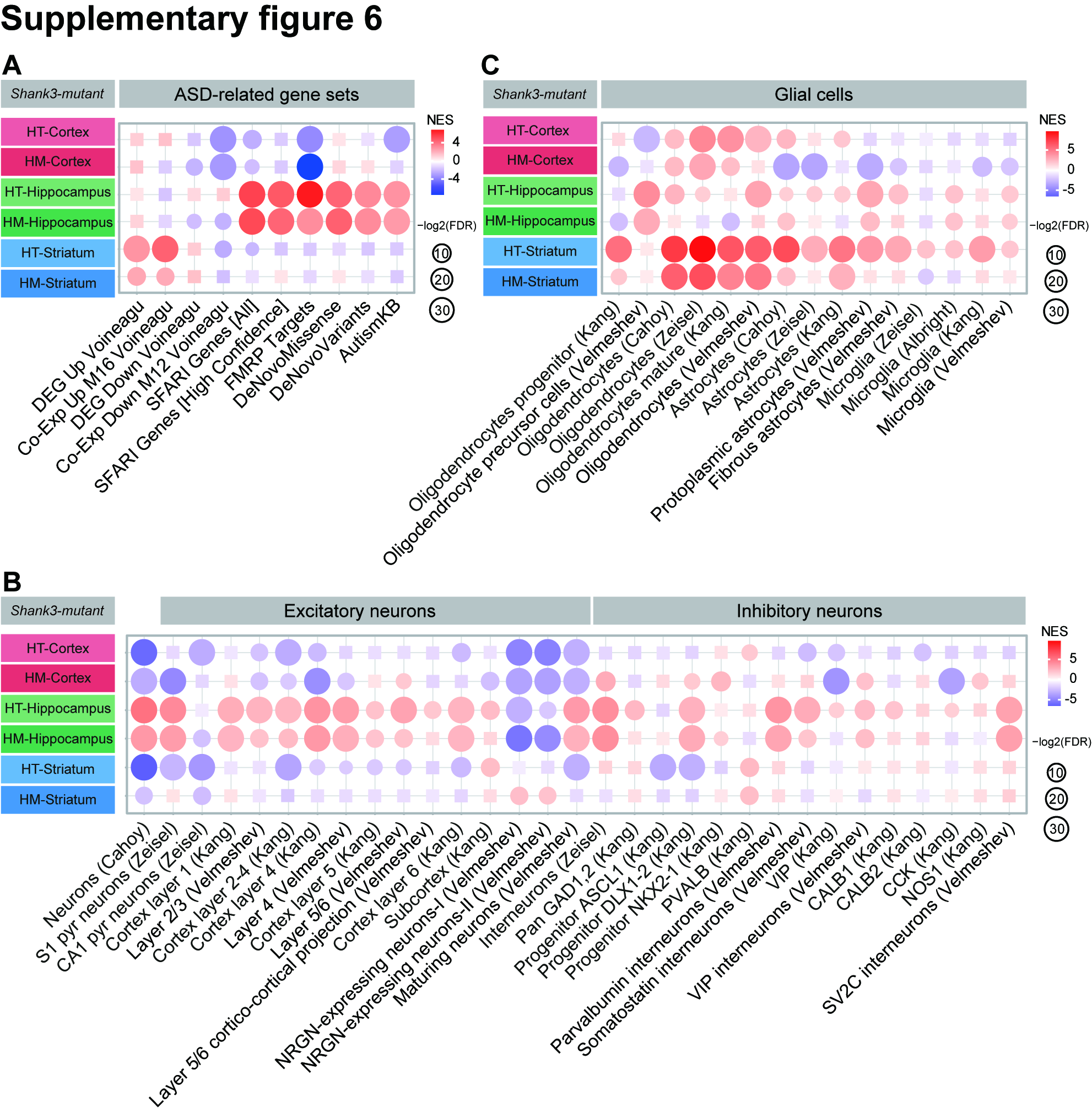

Supplement: Supplementary Figure 6 — ASD-related patterns in the transcripts from the cortex, hippocampus, and striatum of Shank3-HT and Shank3-HM mice. (A–C) The GSEA results in Figure 5 are shown here again with the insignificantly enriched gene sets indicated by square dots, together with the significant enrichments indicated by circular dots, to show that the insignificant enrichments have generally smaller NES scores compared with those of significant enrichments. [file Image_6.TIF]
